# Supplementary figures and images for: Parallel Tempering with Lasso for model reduction in systems biology
Source: PLoS Comput Biol. 2020 Mar 9;16(3):e1007669. doi: 10.1371/journal.pcbi.1007669 (PMC7082068; doi:10.1371/journal.pcbi.1007669)

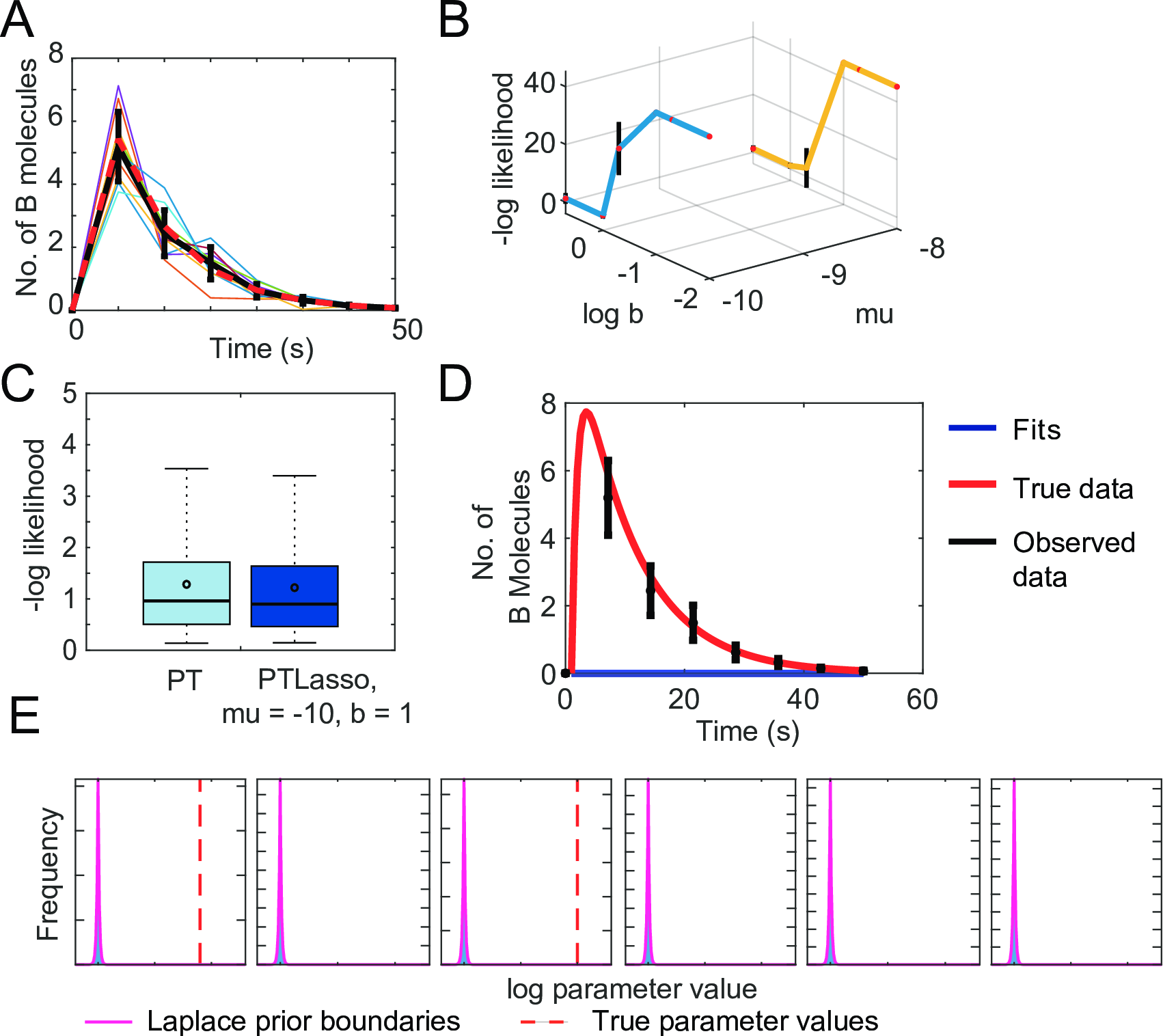

Supplement: S1 Fig — A) Data generated for fitting. Red dashed lines show the model simulation at 8 time points with the true parameter values. Each colored line represents a noisy trajectory obtained by adding Gaussian noise (mean = 0, standard deviation = 30% of the true data value) to the true data. The black error bars show the mean and standard deviation of the 10 repeats, and is the observed data used for fitting. B) Hyperparameter tuning plot showing variation in the negative log likelihood distribution (from 4,000 parameter samples) with μ and b (red points show the mean, and black lines show mean ± standard deviation). The hyperparameters selected (μ = −10, b = 1) provide the most regularization while not substantially increasing the negative log likelihood. C) Comparison of the log likelihood distributions (from 4,000 parameter samples) of the fits with PT and PTLasso (μ = −10, b = 1). Box plots are obtained using a third party MATLAB library, aboxplot*, with outliers not shown. Boxes show data in the 25th–75th percentile and the circles show the mean. D) Example of PTLasso fits (from 4,000 parameter samples) where b is too small (μ = −10, b = 0.1) and the negative log likelihood of the fit is increased, and E) the corresponding parameter distributions (from 400,000 parameter samples). Since the regularization strength was too high, none of the parameters deviated from the prior. *http://alex.bikfalvi.com/research/advanced_matlab_boxplot/. (TIF) [file pcbi.1007669.s001.tif]

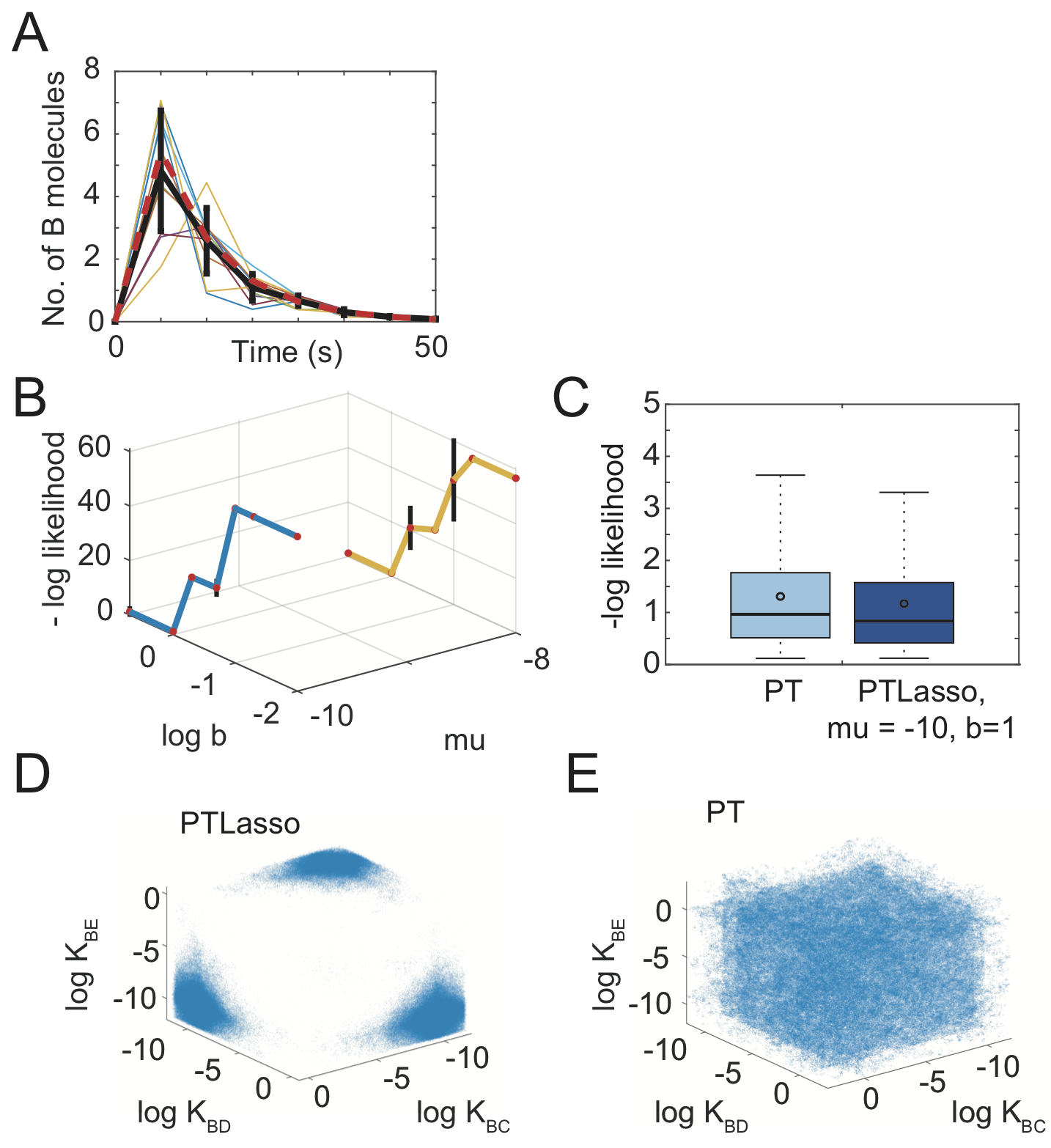

Supplement: S2 Fig — A) Data generated for fitting. Red dashed lines show the model simulation at 8 time points with the true parameter values. Each colored line represents a noisy trajectory obtained by adding Gaussian noise (mean = 0, standard deviation = 30% of the true data value) to the true data. The black error bars show the mean and standard deviation of the 10 repeats, and is the observed data used for fitting. B) Hyperparameter tuning plot showing variation in the negative log likelihood distribution with μ and b (from 7,000 parameter samples, red points show the mean, and black lines show mean ± standard deviation). The hyperparameters selected (μ = −10, b = 1) provide the most regularization while not substantially increasing the negative log likelihood. C) Box plots comparing the log likelihood distribution (from 7,000 parameter samples) obtained with PT and PTLasso for the chosen values of hyperparameters. Box plots are obtained using a third party MATLAB library, aboxplot*, with outliers not shown. Boxes show data in the 25th–75th percentile and the circles show the mean. D). Parameter covariation of the three selected parameters with PTLasso and E) with PT shown as a 3D scatter plot with transparent points (from 700,000 parameter samples). *http://alex.bikfalvi.com/research/advanced_matlab_boxplot/. (TIFF) [file pcbi.1007669.s002.tiff]

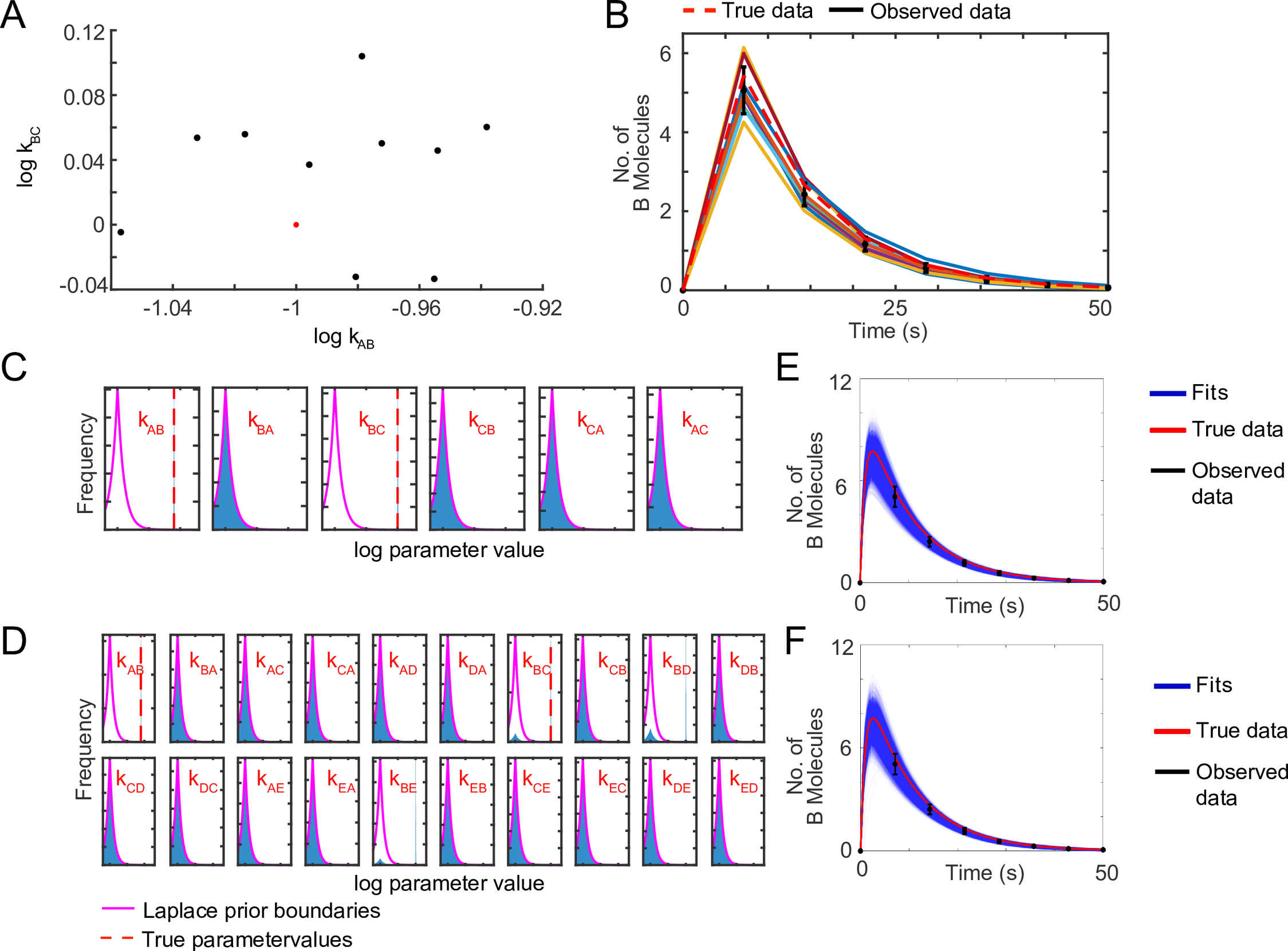

Supplement: S3 Fig — A) Noisy parameter values (black) used to generate the observed data. The log true parameters (red) of the known model were perturbed 10 times with Gaussian noise (mean = 0, standard deviation = 0.05). B) Colored lines show model outputs for each of the 10 noisy parameter sets. The black error bars shows the mean and standard deviation of the colored lines and is the observed data for fitting. Red dashed line shows the model simulation at 8 time points with the true parameter values. C) Frequency histograms showing probability distributions of the parameters (from 800,000 parameter samples) for PTLasso fits of a fully connected three node graph and D) fully connected five node graph. The range of log parameter values on each x-axis is −12 to 3, which covers the full range over which parameters were allowed to vary. The y-axis of each panel is scaled to the maximum value of the corresponding distribution to emphasize differences in shape. The pink lines show the boundaries of the Laplace prior with μ = −10, b = 1, and the dashed red lines in panels for kAB and kBC show the true parameter values. A parameter distribution confined within the Laplace prior boundaries indicates that the parameter is extraneous. E) PTLasso fits to the data for a fully connected three node graph and F) five node graph. Transparent blue lines show ensemble fits (from 8,000 parameter samples, 100 time points per trajectory), red line shows the true data (100 time points), and the black error bars show the mean ± standard deviation of the observed data (8 time points). (TIF) [file pcbi.1007669.s003.tif]

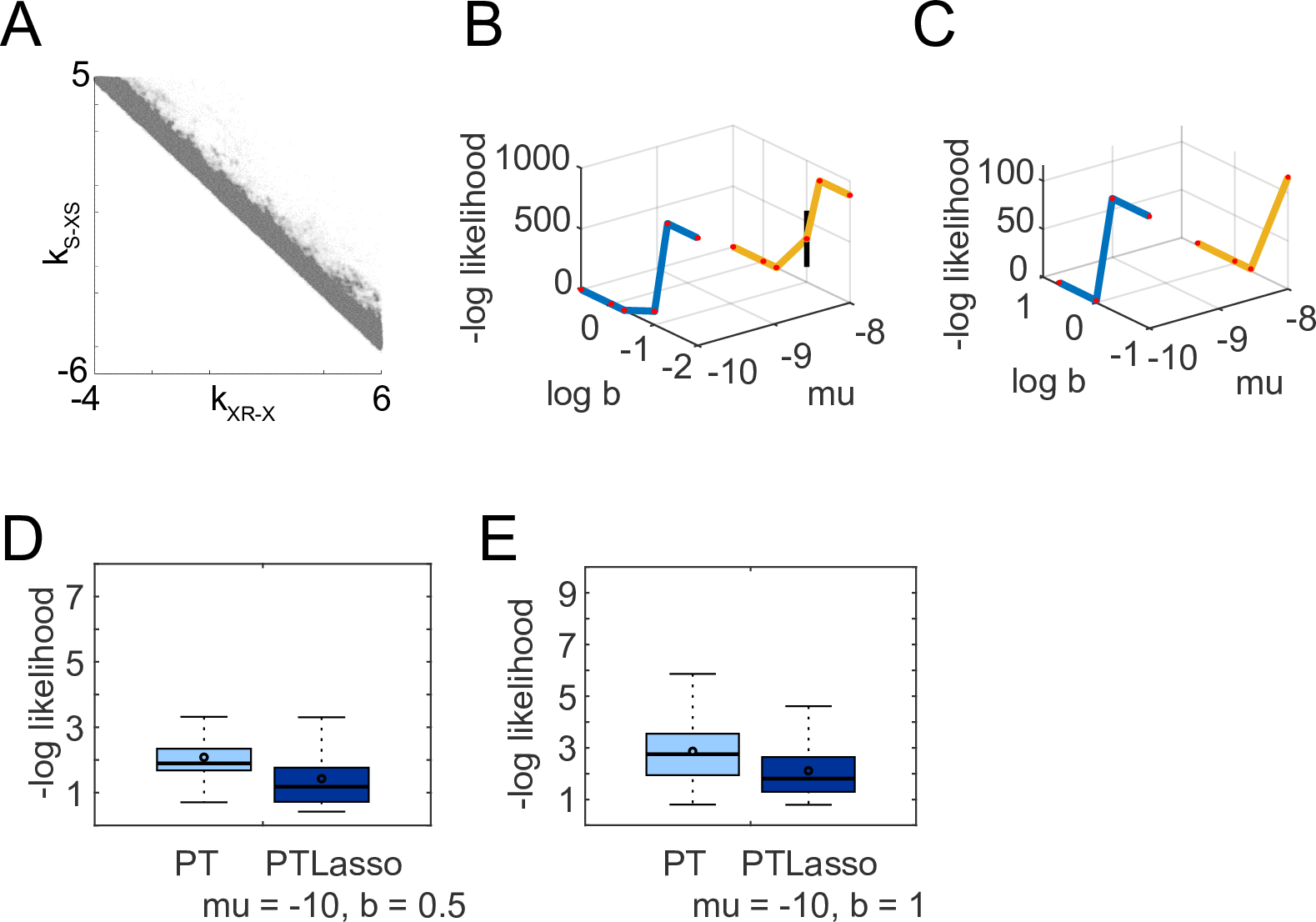

Supplement: S4 Fig — A) Linear correlation of non identifiable parameters in the reduced perfectly adapting model shown as a scatter plot (axes show log parameter values). B) Hyperparameter tuning plot for the linear dose response model and C) the perfectly adapting dose response model. The hyperparameter tuning plot shows variation in the negative log likelihood distribution with μ and b (from 400 parameter samples for the linear dose response model and from 800 parameter samples for the perfectly adapting dose response model. Red points show the mean, and black lines show mean ± standard deviation). The hyperparameters selected (μ = −10, b = 0.5 for linear dose-response and μ = −10, b = 1 for perfectly adapting dose-response) provide the most regularization while not substantially increasing the negative log likelihood. D) Box plots comparing the log likelihood distribution obtained with PT and PTLasso for the chosen values of hyperparameters for the linear dose response model (from 400 parameter samples) and E) the perfectly adapting dose response model (from 800 parameter samples). Box plots are obtained using a third party MATLAB library, aboxplot*, with outliers not shown. Boxes show data in the 25th–75th percentile and the circles show the mean. *http://alex.bikfalvi.com/research/advanced_matlab_boxplot/. (TIF) [file pcbi.1007669.s004.tif]

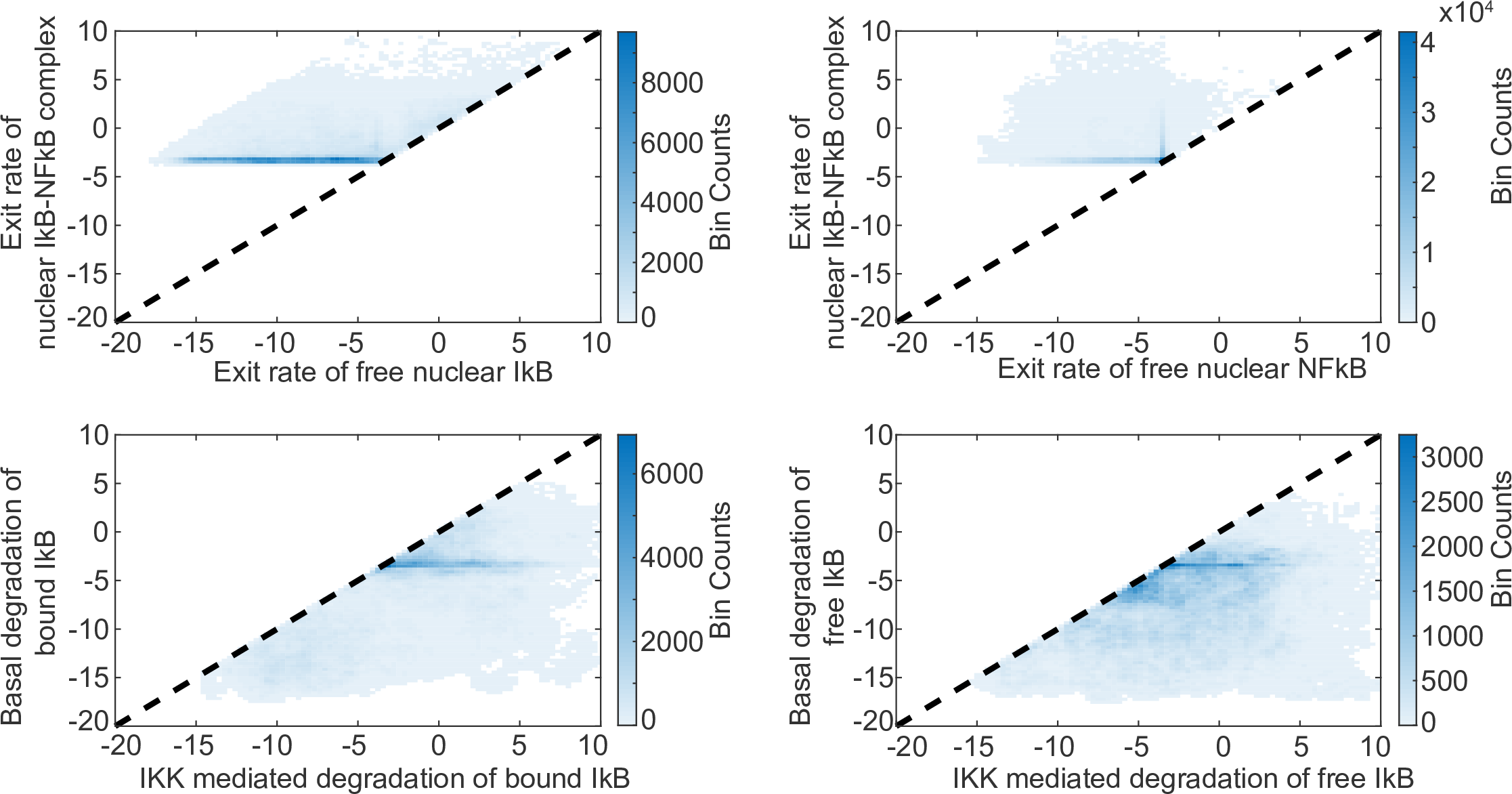

Supplement: S5 Fig — Binned scatter plots (MATLAB function binscatter with 940,000 parameter samples from a PTLasso fit for an NF-κB response to pulsatile TNF stimulation) show the joint distributions for the pairs of parameters for which covariance was constrained during fitting. (TIF) [file pcbi.1007669.s005.tif]

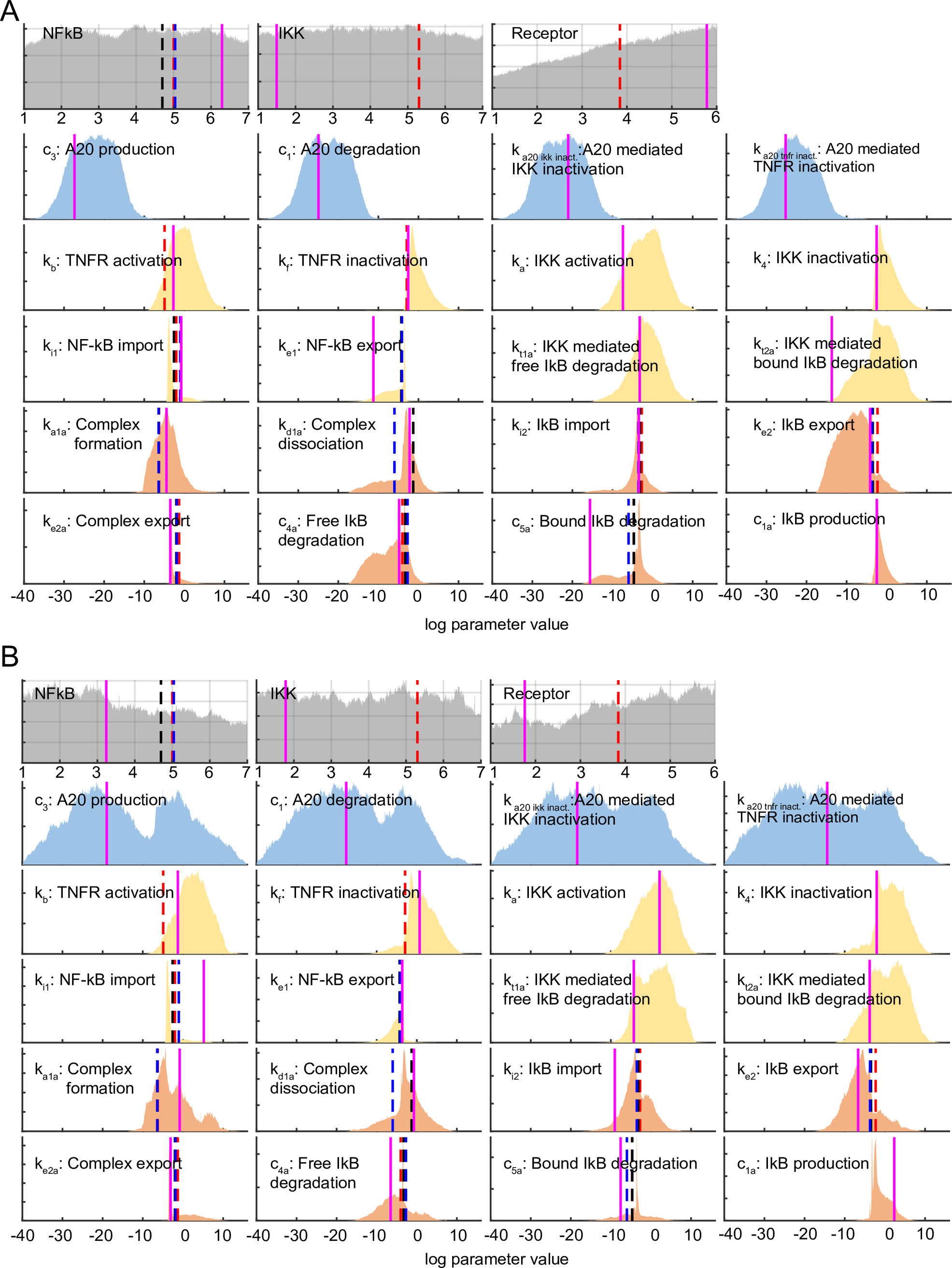

Supplement: S6 Fig — A) with PTLasso and B) with PT. Distributions of total protein abundance parameters and rate constant parameters (from 5,640,000 parameter samples) from the A20 module (blue), Activation module (yellow) and IκB module (orange). All parameters are in logscale. Total protein abundance parameters have uniform priors, and the x-axis range indicates the sampling range. Rate constant parameters are sums of the module penalty parameters and reaction-specific parameters. The pink line corresponds to a best-fit parameter set. The dashed lines correspond to published values of parameters—Pekalski et al. [2] (red), Lee et al. [39] (black), and Kearns et al. [48] (blue). A published parameter value for a model is only included if the corresponding reaction maintained the same structure in both the published and current models. For unit conversions we used the values mentioned in Lee et al. [39], 1μM of NF-κB = 50,000 molecules/cell and applied this to other species in the models. In the parameter labels “import” refers to translocation from the cytoplasm into the nucleus and “export” is the reverse. “Complex” refers to the NF-κB-IκB complex. The y-axis for each panel is scaled from 0 to the maximum value of the distribution to emphasize differences in the shapes of the distributions. (TIF) [file pcbi.1007669.s006.tif]

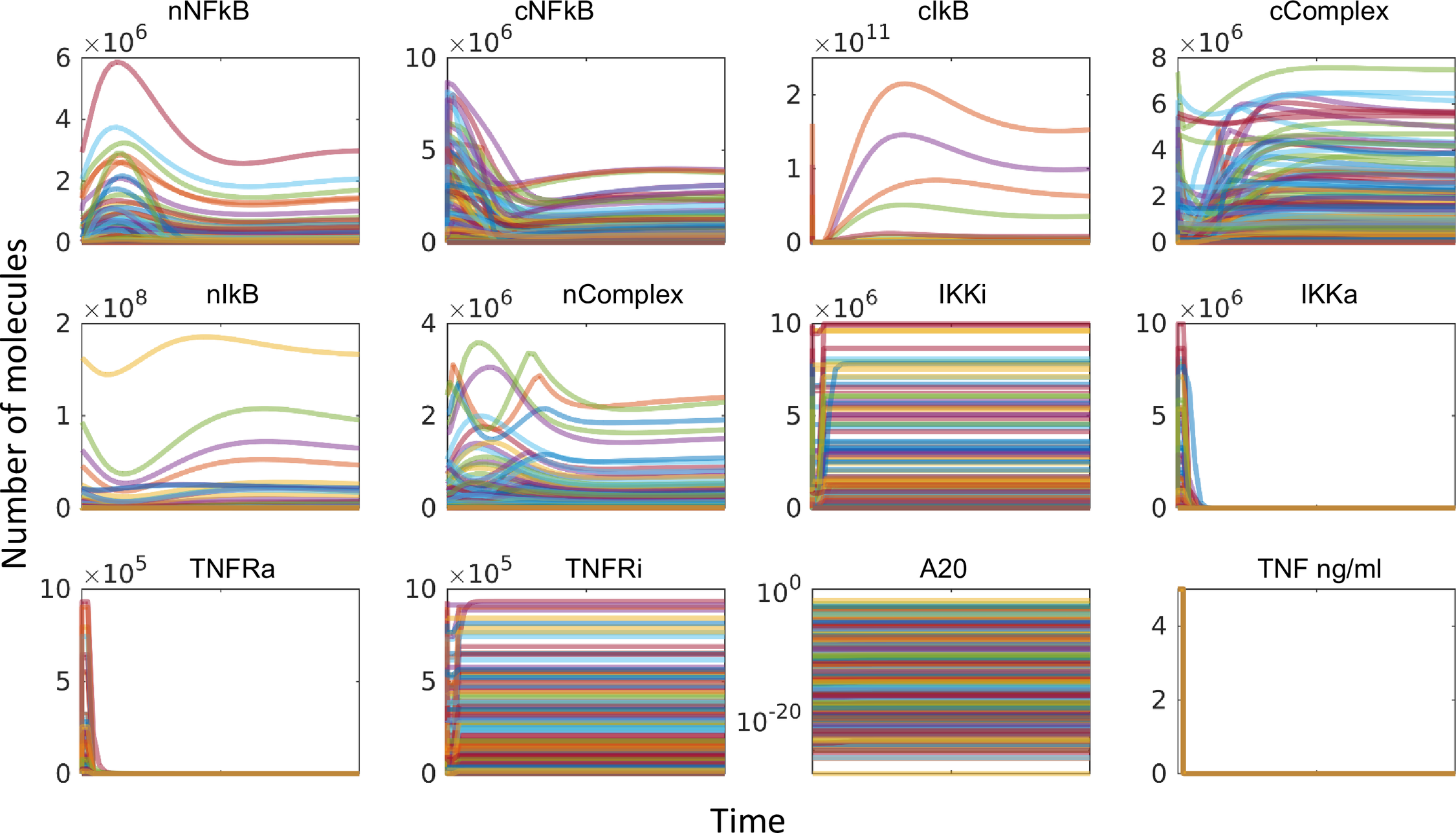

Supplement: S7 Fig — Model predictions for non-fitted variables for a representative NF-κB response to pulsatile TNF stimulation (from 500 parameter samples from one of the PTLasso repeats). Suffix “a” and “i” refer to active and inactive versions of a species respectively. Prefix “c” and “n” refer to cytoplasmic and nuclear versions of a species respectively. “Complex” refers to the NF-κB-IκB complex. Time courses are shown for 4 hours after the initial 5ng/ml TNF stimulation. The TNF concentration is set to 0 at the 5 minute time point. (TIF) [file pcbi.1007669.s007.tif]
