# Supplementary material for: Parallel Tempering with Lasso for model reduction in systems biology
Source: PLoS Comput Biol. 2020 Mar 9;16(3):e1007669. doi: 10.1371/journal.pcbi.1007669 (PMC7082068; doi:10.1371/journal.pcbi.1007669)
Supplement: S1 Table — (PDF) [file pcbi.1007669.s008.pdf]

Table S1. Hard constraints in the NF-κB signaling fits.

Index	Constraint description	Constraint
1	Exit rate of NF-κB-IκB from the nucleus is greater than free NF-κB	ke2a > ke1
2	Exit rate of NF-κB-IκB from the nucleus is greater than free IκB	ke2a > ke2
3	IKK mediated degradation of free cytosolic IκB is greater than the basal rate	kt1a > c4a
4	IKK mediated degradation of NF-κB bound cytosolic IκB is greater than the basal rate	kt2a > c5a
5	At equilibrium there is some NF-κB in the nucleus	$\frac{\text{nNF}\kappa\text{B}_{eq} + \text{nNF}\kappa\text{B} - \text{I}\kappa\text{B}_{eq}}{\text{TotalNF}\kappa\text{B}}$ $\in$ [0.05,0.5]
6	At least 1 TNFR molecule/cell is activated	min(TNFRa) > 1
7	At least 1 IKK molecule/cell is activated	min(IKKa) > 1
8	Equilibrium check before TNF stimulation	Rate of change for each model species < 1e-4 molecules/5e5 seconds
