# Supplementary material for: Parallel Tempering with Lasso for model reduction in systems biology
Source: PLoS Comput Biol. 2020 Mar 9;16(3):e1007669. doi: 10.1371/journal.pcbi.1007669 (PMC7082068; doi:10.1371/journal.pcbi.1007669)
Supplement: S3 Table — Parameter distributions are constructed from the lowest temperature chain. (PDF) [file pcbi.1007669.s010.pdf]

Table S3. MPSRF values for parameter distributions from each example shown up to 4 significant digits. Parameter distributions are constructed from the lowest temperature chain.

**Model name, No. of swaps**	**PTLasso**	**PT**
3-node graph, 400,000	1.001	1.002
5-node graph, 700,000	1.011	1.007
Linear Dose-Response, 400,000	1.000	1.001
Perfectly adapting Dose-Response, 800,000	1.012	1.006
NF-κB signaling (pulse stimulus):		
Trajectory 1, 5,640,000	1.074	1.094
Trajectory 2, 5,640,000	1.118	1.200
Trajectory 3, 5,640,000	1.129	1.014
NF-κB signaling (continuous stimulus) 3,200,000	1.069	N/A
