# Supplementary material for: Parallel Tempering with Lasso for model reduction in systems biology
Source: PLoS Comput Biol. 2020 Mar 9;16(3):e1007669. doi: 10.1371/journal.pcbi.1007669 (PMC7082068; doi:10.1371/journal.pcbi.1007669)
Supplement: S4 Table — M is the number of independent chains that are combined for each group and N is the total number of swaps. The length of each chain is N/M. Energy distributions are constructed from the lowest temperature chain. (PDF) [file pcbi.1007669.s011.pdf]

Table S4. PSRF to show convergence of energy distributions when combining PT or PTLasso chains for the NF-κB signaling fit with pulsatile TNF stimulation. M is the number of independent chains that are combined for each group and N is the total number of swaps. The length of each chain is N/M. Energy distributions are constructed from the lowest temperature chain.

**Trajectory index**	**PTLasso group 1, M=6, N=5,640,000**	**PTLasso group 2, M=6, N=5,640,000**	**PT group 1, M=6, N=5,640,000**	**PT group 2, M=6, N=5,640,000**
1	1.041	1.016	1.049	1.021
2	1.139	1.134	1.011	1.003
3	1.028	1.009	1.001	1.003
