# Supplementary material for: Parallel Tempering with Lasso for model reduction in systems biology
Source: PLoS Comput Biol. 2020 Mar 9;16(3):e1007669. doi: 10.1371/journal.pcbi.1007669 (PMC7082068; doi:10.1371/journal.pcbi.1007669)
Supplement: S5 Table — M is the number of independent chains that are combined for each group and N is the total number of swaps. The length of each chain is N/M. Energy distributions are constructed from the lowest temperature chain. (PDF) [file pcbi.1007669.s012.pdf]

Table S5. PSRF to show convergence of energy distributions when combining PTLasso chains for the NF-κB signaling fit with continuous TNF stimulation. M is the number of independent chains that are combined for each group and N is the total number of swaps. The length of each chain is N/M. Energy distributions are constructed from the lowest temperature chain.

**PTLasso group 1, M=8, N=3,200,000**	**PTLasso group 2, M=8, N=3,200,000**
1.113	1.063
