# Supplementary material for: Parallel Tempering with Lasso for model reduction in systems biology
Source: PLoS Comput Biol. 2020 Mar 9;16(3):e1007669. doi: 10.1371/journal.pcbi.1007669 (PMC7082068; doi:10.1371/journal.pcbi.1007669)
Supplement: S6 Table — (PDF) [file pcbi.1007669.s013.pdf]

Table S6. Step acceptance rates for the lowest temperature chain for each example.

**Model name, No. of swaps**	**PTLasso (repeat 1, 2)**	**PT (repeat 1, 2)**
3-node graph, 400,000	0.24, 0.25	0.24, 0.25
5-node graph, 700,000	0.24, 0.23	0.24, 0.26
Linear Dose-Response, 400,000	0.24, 0.22	0.24, 0.24
Perfectly adapting Dose-Response, 800,000	0.24, 0.24	0.25, 0.25
NF-κB signaling (pulse stimulus), 5,640,000		
Trajectory 1	0.26, 0.26	0.23, 0.22
Trajectory 2	0.26, 0.25	0.45, 0.26
Trajectory 3	0.24, 0.25	0.26, 0.22
NF-κB signaling (continuous stimulus), 3,200,000	0.28, 0.27	N/A
