# Supplementary material for: Parallel Tempering with Lasso for model reduction in systems biology
Source: PLoS Comput Biol. 2020 Mar 9;16(3):e1007669. doi: 10.1371/journal.pcbi.1007669 (PMC7082068; doi:10.1371/journal.pcbi.1007669)
Supplement: S7 Table — (PDF) [file pcbi.1007669.s014.pdf]

Table S7. Swap acceptance rates for the two lowest temperature chains for each example.

**Model name, No. of swaps**	**PTLasso (repeat 1, 2)**	**PT (repeat 1, 2)**
3-node graph, 400,000	0.24, 0.33	0.25, 0.35
5-node graph, 700,000	0.33, 0.47	0.32, 0.26
Linear Dose-Response, 400,000	0.26, 0.97	0.24, 0.24
Perfectly adapting Dose-Response, 800,000	0.17, 0.37	0.25, 0.26
NF-κB signaling (pulse), 5,640,000		
Trajectory 1	0.28, 0.25	0.19, 0.25
Trajectory 2	0.17, 0.17	0.29, 0.32
Trajectory 3	0.16, 0.16	0.26, 0.28
NF-κB signaling (continuous), 3,200,000	0.22, 0.22	N/A
