# Supplementary material for: Parallel Tempering with Lasso for model reduction in systems biology
Source: PLoS Comput Biol. 2020 Mar 9;16(3):e1007669. doi: 10.1371/journal.pcbi.1007669 (PMC7082068; doi:10.1371/journal.pcbi.1007669)
Supplement: S9 Table — The “parameters” column specifies the forward and reverse rate constant pair. True parameters are shown in red, and parameters of the inferred alternate reduced models are shown in blue. All the reactions follow mass action kinetics. First order reaction rate constants are in units of s−1. Second order reaction rate constants are in units of molecule−1s−1. (PDF) [file pcbi.1007669.s016.pdf]

Table S9. Reactions in fully connected five node network. The "parameters" column specifies the forward and reverse rate constant pair. True parameters are shown in red, and parameters of the inferred alternate reduced models are shown in blue. All the reactions follow mass action kinetics. First order reaction rate constants are in units of s^{-1}. Second order reaction rate constants are in units of $molecule^{-1}s^{-1}$.

**Reaction**	**Parameters**	**Reaction**	**Parameters**
A $\longleftrightarrow$ B	k_{AB}, k_{BA}	C $\longleftrightarrow$D	k_{CD}, k_{DC}
A $\longleftrightarrow$ C	k_{AC}, k_{CA}	A $\longleftrightarrow$ E	k_{AE}, k_{EA}
A $\longleftrightarrow$ D	k_{AD}, k_{DA}	B $\longleftrightarrow$ E	k_{BE}, k_{EB}
B $\longleftrightarrow$ C	k_{BC}, k_{CB}	C $\longleftrightarrow$ E	k_{CE}, k_{EC}
B $\longleftrightarrow$ D	k_{BD}, k_{DB}	D $\longleftrightarrow$ E	k_{DE}, k_{ED}
