# Supplementary material for: Parallel Tempering with Lasso for model reduction in systems biology
Source: PLoS Comput Biol. 2020 Mar 9;16(3):e1007669. doi: 10.1371/journal.pcbi.1007669 (PMC7082068; doi:10.1371/journal.pcbi.1007669)
Supplement: S10 Table — The “parameters” column specifies the forward and reverse rate constant pair. All the reactions follow mass action kinetics. First order reaction rate constants are in units of s−1. Second order reaction rate constants are in units of molecule−1s−1. (PDF) [file pcbi.1007669.s017.pdf]

Table S10. Prior network comprising linear dose-response model reactions and adaptive dose-response model reactions. The "parameters" column specifies the forward and reverse rate constant pair. All the reactions follow mass action kinetics. First order reaction rate constants are in units of s^{-1}. Second order reaction rate constants are in units of molecule^{-1}s^{-1}.

**Reaction**	**Parameter(s)**
S $\longrightarrow$ S + R	k_{S-RS}
X + R $\longrightarrow$ X	k_{XR-X}
S $\longrightarrow$ S + X	k_{S-XS}
X $\longrightarrow$ 0	k_{X-0}
R $\longleftrightarrow$ 0	k_{R-0}, k_{0-R}
