# Supplementary material for: Parallel Tempering with Lasso for model reduction in systems biology
Source: PLoS Comput Biol. 2020 Mar 9;16(3):e1007669. doi: 10.1371/journal.pcbi.1007669 (PMC7082068; doi:10.1371/journal.pcbi.1007669)
Supplement: S11 Table — The “parameters” column specifies the forward and reverse rate constant pair. All the reactions follow mass action kinetics. First order reaction rate constants are in units of s−1. Second order reaction rate constants are in units of molecule−1s−1 except kb which is in units of (ng/ml)−1s−1. (PDF) [file pcbi.1007669.s018.pdf]

Table S11. Reactions in NF-κB signaling model. The "parameters" column specifies the forward and reverse rate constant pair. All the reactions follow mass action kinetics. First order reaction rate constants are in units of s^{-1}. Second order reaction rate constants are in units of $molecule^{-1}s^{-1}$ except k_b which is in units of $(ng/ml)^{-1}s^{-1}$.

**Reaction**	**Parameter(s)**	**Module**
TNFR activation		
TNFR(inactive) + TNF $\longrightarrow$ TNFR(active) + TNF	k_b	Activation
TNFR inactivation		
TNFR(active) $\longrightarrow$ TNFR(inactive)	k_f	Activation
IKK activation		
IKK(inactive) + TNFR(active) $\longrightarrow$ IKK(active) + TNFR(active)	k_a	Activation
IKK inactivation		
IKK(active) $\longrightarrow$ IKK(inactive)	k_4	Activation
Reversible cytoplasmic NF-κB-IκB complex formation		
NF-κB (cytoplamic) + IκB (cytoplasmic) $\longleftrightarrow$ NF-κB-IκB (cytoplasmic)	k_{a1a}, k_{d1a}	IκB
Reversible nuclear NF-κB-IκB complex formation		
NF-κB (nuclear) + IκB (nuclear) $\longleftrightarrow$ NF-κB-IκB (nuclear)	$3.3^* \times k_{a1a}$, k_{d1a}	IκB
IKK mediated cytoplasmic free IκB degradation		
IKK(active) + IκB (cytoplasmic) $\longrightarrow$ IKK(active)	k_{t1a}	Activation
IKK mediated cytoplasmic bound IκB degradation		
IKK(active) + NF-κB-IκB (cytoplasmic) $\longrightarrow$ IKK(active) + NF-κB (cytoplasmic)	k_{t2a}	Activation
Basal cytoplasmic free IκB degradation		
IκB (cytoplasmic) $\longrightarrow$ 0	c_{4a}	IκB
Basal cytoplasmic bound IκB degradation		
NF-κB-IκB (cytoplasmic) $\longrightarrow$ NF-κB (cytoplasmic)	c_{5a}	IκB
NF-κB shuttling		
NF-κB (cytoplasmic) $\longleftrightarrow$ NF-κB (nuclear)	k_{i1},k_{e1}	Activation
IκB shuttling		
IκB (cytoplasmic) $\longleftrightarrow$ IκB (nuclear)	k_{i2},k_{e2}	IκB
Linear production of IκB		
NF-κB (nuclear) $\longrightarrow$ NF-κB (nuclear) + IκB (cytoplasmic)	c_{1a}	IκB
Export of NF-κB-IκB complex from the nucleus		
NF-κB-IκB (nucleus) $\longrightarrow$ NF-κB-IκB (cytoplasmic)	k_{e2a}	IκB
Linear production of A20		
NF-κB (nuclear) $\longrightarrow$ NF-κB (nuclear) + A20	c_1	A20
A20 mediated inactivation of IKK		
A20 + IKK(active) $\longrightarrow$ A20 + IKK(inactive)	$k_{\text{a20-ikk-inact}}$	A20
A20 mediated inactivation of TNFR		
A20 + TNFR(active) $\longrightarrow$ A20 + TNFR(inactive)	$k_{\text{a20-tnfr-inact}}$	A20
A20 degradation		
A20 $\longrightarrow$ 0	c_3	A20

* 3.3 is a volume correction factor [1, 2].

References

[1] Ashall L, Horton CA, Nelson DE, Paszek P, Harper CV, Sillitoe K, et al. Pulsatile Stimulation Determines Timing and Specificy of NF-kB–Dependent Transcription. Science. 2009;324(5924):242–247. doi:10.1126/science.1164860.

[2] Lee REC, Walker SR, Savery K, Frank DA, Gaudet S. Fold change of nuclear NF-κB determines TNF-induced transcription in single cells. Molecular Cell. 2014;53(6):867–879. doi:10.1016/j.molcel.2014.01.026.
