# Supplementary material for: Parallel Tempering with Lasso for model reduction in systems biology
Source: PLoS Comput Biol. 2020 Mar 9;16(3):e1007669. doi: 10.1371/journal.pcbi.1007669 (PMC7082068; doi:10.1371/journal.pcbi.1007669)
Supplement: Attachment — Submitted filename: cover_letter_resubmission_PCB.pdf [file pcbi.1007669.s019.pdf]

Dear Drs. Finley and Lengauer,

We are pleased to submit our revision of the manuscript titled “Parallel Tempering with Lasso for Model Reduction in Systems Biology” (PCOMPBIOL-D-19-01641), which we previously submitted for publication in *PLOS Computational Biology*. We appreciate the constructive feedback we have received both from you and the three anonymous reviewers. Their comments encouraged us to revise portions of the text and add extra test results to check the robustness of the method with a different noise model. We have highlighted all the changes in the manuscript, and in our replies we refer to the specific line numbers in the text that address the specific concerns of the reviewers. We believe that our revisions have greatly improved the manuscript and hope that you and the reviewers will now find in suitable for publication in the journal. We look forward to your reply.

Summary of changes:

The only changes that were not requested by the reviewers were minor textual changes and changing the “group lasso” terminology to “grouped lasso”. We realized that in the literature “group lasso” already referred to a specific kind of L2 loss function that was distinct from our approach that applies the L1 penalty on groups of parameters.

All changes are shown in blue for the convenience of the reviewers.

The figures that have changed are Fig. 4 (grouped lasso with NFkB), Fig. S3 (PTLasso with Gaussian noise added to the parameters) and Fig. S6 (Posterior probability distributions for NFkB parameters with and without lasso).

The new tables are Tables S5, S8-S11.

Pointwise responses to the comments to the authors:

Reviewer #1: In this manuscript, Gupta, Lee, and Faeder describe a method for identifying key kinetic modules associated with more complicated mechanistic models using a Bayesian parameter estimation approach coupled with Lasso regularization to reduce the number of parameters. In terms of the review criteria:

Originality - yes, the work appears to be original as it incorporates Lasso regularization. Of note, Klinke and Finley used a Bayesian parameter estimation approach coupled with time-scale separation for model reduction (Klinke and Finley Biotech Prog 2012).

Significant biological and/or methodological insight - methods that enable one to extract some biological insight from a more complicated mechanistic model are helpful. This one uses Lasso regularization as a new aspect.

We thank the reviewer for acknowledging that our use of Lasso regularization is original and may be useful for deriving biological insights. and also for pointing out this reference on using

time-scale separation for model reduction. We added the reference, which is now cited on line 20 along with the other time-scale based methods that we cite for model reduction.

Rigorous methodology -

- Couple of concerns:

1. The authors choose to incorporate a Lasso regularization approach to minimize the number of parameters in the model. Given that parameters in system biology models may not be tightly constrained and may exhibit correlation with other parameters, a Lasso approach only chooses one parameter in a set of correlated parameters. The choice is a bit arbitrary. So what is it to say if the Lasso chooses another parameter in the correlated set, would the conclusions be different?

We thank the reviewer for raising this very important point of finding multiple regularization solutions where present.

In response to this comment we more thoroughly investigated our five-node example, which was a case where multiple minimal sets of parameters (or mechanisms) can fit the data, corresponding to the reactions A->B->C, A->B->D, and A->B->E. PTLasso found all three mechanisms, but the relative probabilities of the mechanisms (which should be equal due to symmetry) showed differences in the relative heights of the posterior distributions for k_{BC}, k_{BD} and k_{BE} (see Fig. 2D and Fig. S3D).

While Bayesian methods are global and in theory should find the correct probabilities of all local minima in a multimodal landscape, limitations of sampling with MCMC methods make this difficult to achieve in practice, even when metrics such as the PSRF or MPSRF are below the suggested convergence thresholds (as was the case for this 5-node example). We have added a paragraph to the discussion (lines 409-417) that makes these caveats to the reader, and once again would like to thank the reviewer for drawing our attention to this important problem.

2. Given the common presence of parameter non-identifiability in these types of models, convergence applied to the parameters doesn't really make sense, while applied to the model predictions does. Once the chains have converged, they are sampling the posterior distribution. It seems here, the authors only ran the MCMC chains until they converged and then used the unconverged segments. That's not correct.

We agree with the reviewer that assuring chain convergence is critical on all Bayesian parameter estimation approaches. We apologize for not being clearer in describing our procedures for checking convergence and sampling from converged chains.

For all PT and PTLasso chains, we discard an initial portion of the chain as burn-in. The posterior parameter distributions are constructed from the remaining portions of the chains. We obtain the posterior parameter distributions twice (by repeatedly running PT or PTLasso) and

use the potential scale reduction factor (PSRF) and its multivariate extension (MPSRF) to determine convergence of the parameter distributions.

While convergence of individual parameter values may not make sense because of issues of parameter identifiability, the PSRF and MPSRF are standard metrics to check for convergence of parameter distributions from MCMC methods. We have changed the text to say convergence of parameter distributions instead of convergence of parameters and we thank the reviewer again for raising this important point.

3. It's unclear to me about the point of swapping chains. In this implementation, the proposed step only depends on the current point. Swapping chains just artificially inflates within chain variance and reduces between chain variance such that the Gelman-Rubin PSRF would be reduced, suggesting early convergence. Of note, the majority of the time is spent solving ODEs - so figure out a way to speed up solving the ODEs, especially stiff ones.

We thank the reviewer for raising these important points and apologize for not being clear about the various other bottlenecks for MCMC methods such as ODE integration time in the text.

We would like to clarify that while the higher temperature chains are effectively sampling shallower energy landscapes, the lowest temperature chain is always sampling from the true energy landscape. We have now highlighted this line in the Parallel Tempering section in Methods. All the parameter sets drawn from the converged lowest temperature chain fit the data.

While we have not analyzed the effect of swapping on the chain variances, several previous papers (Earl and Deem, J. Phys. Chem. 2005, Gupta et al., PDP 2018, Zhang et al., Computers and Chem. Eng. 2018) have described the parallel tempering approach as an efficient sampling strategy. The purpose of swapping is to allow high temperature chains that sample parameter space more broadly to exchange parameter sets with lower temperature chains, allowing them to more easily escape local minima.

While we agree that ODE integration is a bottleneck, speeding up the integration is beyond the scope of this manuscript. For efficiency we currently perform the ODE integration in C using highly optimized CVODE libraries, which are called using MATLAB’s Mex interface. We have added a line in the text to clarify this (Lines 248-249).

Importance to researchers in the field - Yes, this may be helpful to researchers in the field to reduce the complexity of models based on the data available.

We thank the reviewer for concluding that PTLasso will help researchers reduce their models based on available data.

Other minor comments:

Unclear how PT is better than adaptive MCMC methods, which are much faster than plain MCMC.

We thank the reviewer for this important question. We had not sufficiently clarified this but our implementation of PT is also adaptive and the step sizes and temperatures are allowed to change during a burn-in phase. We refer to our previous paper Gupta et al., 2018, PDP in the Software section for details of the implementation.

In Gupta et al. 2018, PDP, we compared Parallel Tempering with the Metropolis-Hastings algorithm, both implemented with adaptive step sizes. We found for a range of six biological models of increasing complexity that Parallel Tempering converged faster.

Unclear how noise properties impacts results using toy model. Experimentally, noise distribution may be constant (i.e., independent of the true value) or may be inversely proportional to the true value (RNAseq data is like that).

We agree with the reviewer that the impact of the noise properties on the synthetic data fits is an important avenue for investigation.

To more accurately mimic cell-to-cell variability in biological signaling systems, we tested a second noise model where the data was generated from a known model with noisy parameter values. In keeping with the reviewer's suggestion we added constant (independent of the true value) Gaussian noise (mean=0, standard deviation=0.05) to the log parameters 10 times and collected 10 noisy model outputs. As previously, the mean and standard deviation of the noisy model outputs formed the observed data for fitting.

Fitting to noisy data generated by adding constant Gaussian noise to the parameters did not qualitatively change the results of PTLasso when reducing a fully connected three-node or five-node network. The new results are presented in Fig S3 and described in lines 301-309.

Reviewer #2: This paper presents a novel computational method (PTLasso) for identifying reduced mathematical models of signaling networks. By searching for reduced models that can fit temporal experimental data, the method yields information on critical pathway components, modules, or parameters that are necessary to explain observed data. In my opinion, this paper addresses major needs in the area of quantitative systems biology/mathematical biology: first, by seeking reduced models it addresses the issue of overfitting (which is a particular challenge in cell signaling, where models based on prior knowledge are becoming increasingly complex, even while very limited information about parameter values is available); second, it addresses the issue of how to interpret model fits (by revealing which aspects of a model are actually necessary to reproduce experimental observations). Overall I believe this paper makes a significant contribution to cell signaling and other areas of quantitative biology, which should be published and is a good fit for PLOS Comp. Bio. The methodological approach is solid.

We thank the reviewer very much for their positive comments and for noting that PTLasso is addressing important needs in the mathematical biology community in avoiding overfitting of

complex models to limited data and also providing a way to interpret model fits by isolating parts of the model that are required for experimental observations.

However, there are a number of issues with the presentation that should be addressed before publication, detailed below.

1) Throughout the paper, the mathematical models are presented only graphically. If I understand correctly, all models are treated as ODEs with added Gaussian noise.

I think it would be helpful to include in the Supplement the explicit equations for all models.

This would aid reproducibility and help prevent potential confusion, for example:

a) I was initially confused about the usage of both capital Ks and lower-case ks to denote kinetic rate parameters, since capital Ks are typically associated with equilibrium constants (i.e., ratios of kinetic parameters). I would recommend using only lower-case ks (e.g., in Figures 1 and 2), but regardless, showing the full models would clarify any potential confusion about what the parameters mean.

b) In the caption of Figure 3 model description, it reads “Solid lines show species conversions and dashed lines show influences”. I think I know what is meant by this in terms of mass-action kinetics, but I’m not sure it would be universally understood. Again, the full equations would prevent any confusion.

We thank the reviewer for these important points and apologize for our oversight. Accordingly we have made the following changes that we believe address the reviewer’s concerns about the readability of the manuscript and presentation of the models:

(1) We have added tables showing the reactions for all the models used in the paper. We have now added Tables S8-11 that describe the reactions in each of the models used.
(2) We have changed all the capital Ks to lower case k whenever describing rate constants.
(3) We have clarified the meaning of “influence” by adding the following sentence in the captions for Fig 3 and 4: “ … influences, where a species influences the rate of the corresponding reaction without being consumed”.

2) In the captions of Figs. 1-3, the parameter values are reported without any units. The units should be clarified (I assume they are per-second rate constants, since the simulated time trajectories are presented in seconds)

We thank the reviewer for catching this omission. We have added the units to all the captions for Figs 1-3 (either per-second or per-molecule per-second).

3) It would be helpful if the x-axis could be labeled with numeric values for the parameter plots (e.g., Fig 1C, Fig 2C and D, etc.). With the plots unlabeled, it’s very difficult to figure out where the prior was centered (the value of mu) and how that value compares to the true parameter values. I realize this is a challenge because of space limitations. At leat, it would be helpful to

clarify the chosen values of mu and b directly in the figure and/or figure caption, along with the values of the true parameters for comparison.

We agree with the reviewer that the information about the center of the prior (mu) and the true parameter values is important for interpreting the results, however the space constraints made it difficult to show these numbers in the figures. We have however now added this information -- mu, b for the PTLasso fits, the range of the x-axis and the values of the true parameters -- to the captions in Fig 1-4.

4) Throughout the paper, it is unclear what is meant by the number of samples. For example, in Figure 1B, the curves represent “4e3 samples” and in Figure 1C, the distributions show “4e5 PT samples” (is it the same number for both PT and PTLasso?). Why is the number of samples shown in 1B and 1C different? I had a hard time understanding (1) what does one sample mean, in terms of the MCMC procedure described. Does one sample = one run of many iterations? If so, is it subject to the convergence criterion described in Methods? (2) What determines the number of samples used, which is different for different models?

We thank the reviewer for raising this important point and apologize for causing confusion. A “sample” refers to a parameter set drawn from the lowest temperature chain for either PT or PTLasso.

We have added the following text to clarify this: “we often refer to a parameter set obtained from the lowest temperature chain at a particular swap as a sample. Ensemble fits are shown by sub-sampling parameter sets from the lowest temperature chain” (Lines 133-135). For clarity we have also changed the text in all the captions to say “parameter samples” instead of “PT samples” or “PTLasso samples”

Frequency histograms are shown for all the samples in a converged chain (e.g. 4e5 samples in Fig 1C). While this number is different for different models, it corresponds to the number of samples used to determine convergence for a particular example (Tables S2 and S3).

To save on computational time we show simulated fits by evenly subsampling from the chain. In Fig 1B, simulated fits are shown for every 100th parameter sample from the converged portion of the lowest temperature chain, resulting in 4e3 simulated fits.

5) The results for NFkB signaling seem central to the paper, since the authors here show an application to real-world experimental data. I think the presentation of Figure 4 and corresponding section of Results could be improved:

We thank the reviewer for their suggestions on improving this section of the paper.

a) It’s unclear to me why Fig. S7 is in the supplement, and not included in the main text (perhaps as part of Figure 4, or as a stand-alone figure). It seems like an important result that demonstrates the utility of the method: that PTLasso identifies the A20 network module as being necessary to reproduce the nNFkB trajectories under continuous stimulation, whereas it is NOT

necessary to reproduce measurements from short-pulse stimulated cells. From a biological standpoint, it suggests that cells may engage negative feedback loops (or other types of modules) only under certain stimulus conditions. This is very interesting!

We agree with the reviewer that the observation that certain signaling modules may be engaged under certain stimuli but not others is indeed very interesting. Accordingly we have now moved Fig. S7 to be part of Fig 4. We have also added information about convergence and step/swap acceptance rates etc. for this example to Tables S2-S3,S5-S7.

b) The presentation in Fig. 4 is confusing, because in 4C results for both PT and PTLasso are shown, whereas in 4B only “PT Fits” are mentioned, but I believe PTLasso is implied.

We apologize for the confusion. The reviewer is correct and we have updated the label to say “PTLasso”

c) In Fig. 4, only the PTLasso penalty parameter distributions are shown, which seems inconsistent in presentation with the rest of the paper, in which distributions of parameters from both PT and PTLasso are directly compared. (And since PT serves as the baseline of comparison throughout the paper, to support the utility of PTLasso). I guess the challenge is that there are so many parameters in the NFkB signaling network. Still, it would be nice to see a visual representation of the output of PT versus PTLasso, especially since it is emphasized that the PT and PTLasso fits are equally good (4C). I was particularly interested in whether the PT fits also hint at some modularity, or do the PT parameters tend to involve all modules? In other words, is PTLasso really necessary to reach the conclusion that the A20 module is not necessary, or can PT alone reach this conclusion, perhaps indirectly? I wonder if there’s a way to show parameter ensembles, somehow lumped by module. Another solution would be to directly show the PT and PTLasso parameter distributions in the Supplement, where space is not limiting (and group the parameters by module). I think this is already done for PTLasso in Fig. S5, where the A20 module parameters tend to be centered around low values (-20 to -30). Do the corresponding plots for PT show higher values and/or broader distributions of A20-module parameters? This would support the idea that PTLasso is really necessary over PT to eliminate the A20 module.

We thank the reviewer for suggesting that we improve our comparison between PT and PTLasso in the NFkB model example. After careful consideration, we have added a representative PT result showing the NFkB fit and penalty parameter distributions to Figure 4. Note that in this case the penalty parameters have uniform priors. We have also added all the PT parameter distributions grouped by module to Fig S6 to compare with results from PTLasso (previously this was Fig S5).

The reviewer’s intuition was correct and the PT fits do show broader distributions for the A20 penalty parameter and A20-module parameters (now mentioned on Line 381). We hope that this will make the advantage of using PTLasso over PT clearer for the readers.

d) I don't think Fig. S5 is ever referred to in the Main Text (I couldn't find it). If I understand, it shows that PTLasso recovers best fit parameter values that are in line with previous estimates. This seems worth describing in Main Text. However, it needs to be clarified which NFkB trajectory these parameters correspond to: continuous or short-pulse stimulation.

We thank the reviewer for noticing that we had missed referring to Fig. S5. We have added this text (in blue) to refer to this figure (now Fig S6):

"The posterior distributions estimated for many of the reaction rate constants showed overlap with values previously reported in the" (Line 383)

We also note that while many of the distributions do show overlap with literature values, they are also very broad extending well into non-biophysical regimes. We believe this is an effect of the broad sampling ranges used when we perform regularization on parameters in log-scale combined with non-identifiability in the model.

6) This might be a minor detail, but in line 144, the authors neglect the normalization constant 1/2b. If I'm not mistaken, this means that the weight of the prior (relative to the likelihood) in the energy function is changing in a way that depends on the hyperparameter b. Is this justified?

We thank the reviewer for this important question. The dependence of the energy function on b (highlighted in the section on Regularization with Lasso) is central to the Lasso idea.

When b is very small, contribution of the regularizing prior to the energy calculation is high compared to the likelihood, whereas if b is very high the contribution of the regularizing prior is low. Our hyperparameter tuning strategy aims to find the smallest value of b that does not reduce the likelihood -- or in other words achieves maximum regularization (the sparsest model), without reducing the quality of the fit (described in lines 233-236 in the "Hyperparameter selection" section).

7) There are some typos in line 113: missing vector notation on theta, and a missing period.

We thank the reviewer for this catch and have corrected these typos.

8) Could the authors clarify the relationship between equations 1 and 2? Is there an explicit equality between p(Y given theta) and L(theta)?

We apologize for not clarifying this earlier -- p(Y|theta) is the normalized L(theta). We have added a line in the text to clarify this (Line 96).

Reviewer #3: The paper by Gupta et al addresses an important problem in computational biology - the calibration of parameters in ODE kinetic models and model reduction. The authors extended their past work on parallel tampering and Bayesian MCMC chains to include Lasso regularization. Regularization is very important in large models and the Lasso framework they use merges very well with the parallel tempering algorithm. Overall I recommend that the paper be published by Plos Comp Bio. I have a few suggestions that I think can improve the paper

without too much effort and I recommend that these will be suggested to the authors so they can decide which to implement.

We thank the reviewer very much for their positive comments on the importance of our work on model calibration with regularization and for recommending our paper for publication.

I did not understand how the authors dealt with parameter units. Parameters have values that have are on an arbitrary scale, some could be order of magnitude different from others. In their MCMC runs, parameter scaling matters in their multivariate proposal distribution (eq 4.). This is especially true if parameter covariance is taken into account. One suggestion (see Yao et al MSB 2016) is to normalize all values related to a reference value using log of the fold difference. There are other ways of addressing this issue and perhaps the authors already do and I missed it. But if they don't I think that they should.

This is a great point raised by the reviewer and we apologize for not clarifying our approach better.

All the parameters are in log-scale mitigating the scaling problem. We previously mentioned this in the "Regularization with Lasso Section -- "For efficiency we perform parameter estimation in log parameter space" (Line 152). For additional clarification we have added another line in the Parallel Tempering section -- "Another way to increase efficiency of sampling for parameters that may be on different scales is to sample in log-space as we have done for all of the examples in this manuscript." (Lines 140-142)

The authors used synthetic data to test their methodology. I think this is a great approach. However, the way they implemented the addition of noise in synthetic data is problematic. The addition of Gaussian white noise is very unrealistic. The majority of variability in biological measurement is not due to instruments where the idea of Gaussian noise is reasonable but from biological variability. An alternative I would suggest is to add parameter variability (that could be gaussian) and run multiple simulations of the ODEs and use that to generate the synthetic data and see how good is model calibration is under these conditions.

We thank the reviewer for drawing our attention to a more biologically relevant noise model as we hope that our approach will be relevant to studying real biological problems.

As per the reviewer's suggestion we added Gaussian noise (mean=0, standard deviation=0.05) to the log parameter values 10 times and collected 10 noisy model outputs. As previously, the mean and standard deviation of the noisy model outputs formed the observed data for fitting. We note that we have also described this in response to Reviewer 1's query about the robustness of the results to various noise models.

Fitting to noisy data generated by adding constant Gaussian noise to the parameters did not qualitatively change the results of PTLasso when reducing a fully connected three-node or five-node network (see Fig S3 and lines 300-309).

In the NFkB model, the authors used both hard and soft constraints. It will be great to know if these were essential, i.e. can the system “work”, create good fits even if these constraints are violated?

We thank the reviewer for this interesting question about the impact of the biologically motivated constraints on the results.

Theoretically, model fits with a hard constraint should be a subset of model fits without the constraint, so lifting the hard constraints should not reduce the quality of the NFkB fit.

With regards mechanisms revealed by regularization -- preliminary analyses indicate that if the constraint that the nuclear NFkB-IkB complex exit rate should be higher than the free nuclear NFkB exit rate (a known property in NFkB signaling) is not applied for the continuous treatment case, a spurious mechanism may be possible where A20 is regularized out but the dominant nuclear species is the bound complex and not free NFkB. However we think that while a systematic investigation of all the biologically motivated constraints would be interesting, it is beyond the scope of this paper.

The authors explain how they tune hyperparameters related to the prior. But I did not see any discussion related to other hyperparameters related to the proposal distribution, the tempering rate, the likelihood function (i.e. the sigma of the residual). Are these less important? Or should they also be tuned?

We thank the reviewer for raising this important point regarding the other hyperparameters, and in response we now provide the code for fitting used for all the computational experiments. This contains the values for all the other hyperparameters used as well as the exact synthetic data with the corresponding values for sigma) https://github.com/RuleWorld/SupplementalMaterials/tree/Gupta2019/Gupta2019. We have posted this link on line 205 of the manuscript.

Since the temperatures and step sizes are adaptive and change during the burn-in phase to ensure good step and swap acceptance rates (Table S3,S7), the initial choice of hyperparameters does not require much tuning. Further details about the algorithm implementation are included in our previous paper, Gupta et al., 2018, PDP.
